# Phosphorylated Tau in Alzheimer’s Disease and Other Tauopathies

**DOI:** 10.3390/ijms232112841

**Published:** 2022-10-25

**Authors:** Priyanka Rawat, Ujala Sehar, Jasbir Bisht, Ashley Selman, John Culberson, P. Hemachandra Reddy

**Affiliations:** 1 Department of Internal Medicine, Texas Tech University Health Sciences Center, Lubbock, TX 79430, USA; 2 Department of Pediatrics, Texas Tech University Health Sciences Center, Lubbock, TX 79430, USA; 3 Department of Family Medicine, Texas Tech University Health Sciences Center, Lubbock, TX 79430, USA; 4 Nutritional Sciences Department, College Human Sciences, Texas Tech University, Lubbock, TX 79409, USA; 5 Department of Pharmacology and Neuroscience, Texas Tech University Health Sciences Center, Lubbock, TX 79430, USA; 6 Department of Neurology, Texas Tech University Health Sciences Center, Lubbock, TX 79430, USA; 7 Department of Public Health, Graduate School of Biomedical Sciences, Texas Tech University Health Sciences Center, Lubbock, TX 79430, USA; 8 Department of Speech, Language, and Hearing Sciences, Texas Tech University Health Sciences Center, Lubbock, TX 79430, USA

**Keywords:** Alzheimer’s disease, dementia, amyloid beta, phosphorylated tau, oxidative stress, tauopathy

## Abstract

Alzheimer’s disease (AD) is the leading cause of dementia in elderly people. Amyloid beta (Aβ) deposits and neurofibrillary tangles are the major pathological features in an Alzheimer’s brain. These proteins are highly expressed in nerve cells and found in most tissues. Tau primarily provides stabilization to microtubules in the part of axons and dendrites. However, tau in a pathological state becomes hyperphosphorylated, causing tau dysfunction and leading to synaptic impairment and degeneration of neurons. This article presents a summary of the role of tau, phosphorylated tau (p-tau) in AD, and other tauopathies. Tauopathies, including Pick’s disease, frontotemporal dementia, corticobasal degeneration, Alzheimer’s disease, argyrophilic grain disease, progressive supranuclear palsy, and Huntington’s disease, are the result of misprocessing and accumulation of tau within the neuronal and glial cells. This article also focuses on current research on the post-translational modifications and genetics of tau, tau pathology, the role of tau in tauopathies and the development of new drugs targeting p-tau, and the therapeutics for treating and possibly preventing tauopathies.

## 1. Introduction

**Figure 1:**
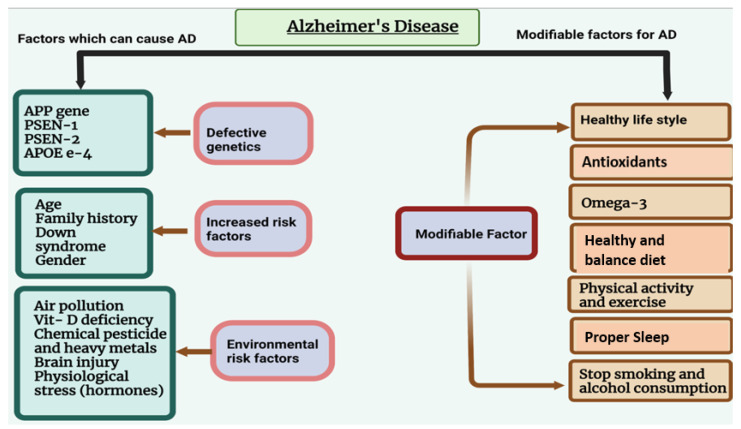
Non-modifiable and modifiable risk factors for Alzheimer’s disease. Non-modifiable factors that may cause AD are age, sex, and defective genetics. Modifiable risk factors (e.g., obesity, smoking, drinking, heart disease, physical inactivity) can play a major role as preventive factors.

Alzheimer’s disease (AD) is the leading common cause of dementia in elderly individuals. AD is characterized by different symptoms, including loss of cognitive behavior and response to the environment, memory loss, and difficulties with thinking, language, and other mental abilities [1]. AD refers to morphological and pathological changes that occur in the brain during the disease process, primarily in the hippocampus and multiple areas of the cortex that control learning and memory. AD typically destroys neurons and their connections in the entorhinal cortex, hippocampus, and cortex responsible for language, behavior, and reasoning [2,3]. Accumulation of amyloid beta and phosphorylated tau within the brain cause disturbances in synaptic function and loss of neuronal cells [3]. AD is caused by both modifiable and non-modifiable risk factors (Figure 1). Table 1 shows the genetics of AD.

In AD and other tauopathies, hyperphosphorylated tau, the formation of paired helical filaments and neurofibrillary tangles are believed to be caused by pathological modification in tau protein that leads to the conformational changes in the structure of tau [4]. However, the role of hyperphosphorylated tau, being the cause or the consequence of tauopathies is still unclear, and more research studies need to be done. Tau proteins are predominantly found in the axonal part of the neuron and are responsible for the stability and assembly of microtubule (MT) protein [5,6]. Tau is also redistributed in soma and dendrites [5]. Recent studies showed that tau is also found in glia and astrocyte [6]. Tau and other microtubule-binding proteins (MAPs) that binds to the MTs are strictly regulated by several factors to ensure the proper dynamics of the system [7]. Once the tau is detached from the MTs, tau accumulates in the neurites and neuronal cell bodies and forms NFTs, which are one of the major pathological features of AD and other tauopathies [8,9]. The deposition of paired helical filaments (PHFs) and neurofibrillary tangles (NFTs) results in the impairment of physiological functions, apoptosis, and neuronal loss, which is reflected as cognitive impairment, and progression in the disease leads to death [4]. Mutation in the tau gene is linked with frontotemporal dementia and parkinsonism, which shows that tau dysfunction could lead to neurodegeneration [10].

A growing body of evidence suggests that oxidative stress is one of the reasons for tauopathies; oxidative stress plays an essential role in tau hyperphosphorylation, polymerization, and tau toxicity [11]. Tau aggregates show different morphological and biochemical features in tauopathies [12]. The objectives of this article are to summarize the role of tau in microtubule structure and function, post-translational changes, and the pathological role of tau in AD and tauopathies. This article also covers the genetics of tau, tau-mitochondria and synaptic nexus, tau-induced inflammation, tau therapeutics, and new tau-based medicines.

**Table 1:** Summary of major genes responsible for Alzheimer’s disease.

Gene	Locus	Pathology of AD	Function	Dysfunction in AD
APP	21q21.1–21q21.3	Early-onset Alzheimer’s disease (EOAD)	Neural plasticity and regulation of synapse formation [13].	Formation of amyloid protein and mitochondrial dysfunction [14].
PSEN-1	14q24.2	Early-onset Alzheimer’s disease (EOAD)	Formation of the catalytic core of γ-secretase complex and proteolytic cleavage of the APP and NOTCH receptor protein [13].	Increased production of amyloid-beta 42.
PSEN-2	1q42.13	Both early-onset and late-onset Alzheimer’s disease.	Cleavage of amyloid-beta.	Deposition of amyloid-beta and neuronal cell death.
APOE-4	19q13.2	Both familial late-onset and sporadic late Alzheimer’s disease.	Distribution and metabolism of cholesterol.	Cerebral glucose hypometabolism (Metabolic dysfunction) [15]. Dysfunction of BBB [16]. Amyloid aggregation and tau hyperphosphorylation

## 2. Microtubule-Associated Protein Tau (MAPT) Structure and Function

The tau protein has six isoforms, and they are produced by alternative splicing of the microtubule-associated protein tau (MAPT) gene. Tau’s physiological function is to stabilize the internal skeleton of neurons in the brain [6]. Since tau is a microtubule-associated protein, microtubules facilitate neuronal cells in maintaining their structure and help in cell division, which is also required for the transportation of material among cells.

*The tau gene and tau isoforms.* Microtubule organization, stability, and polymerization are regulated by microtubule-associated proteins MAP1, MAP2, and tau [17]. The MAPT gene is located on chromosome 17q21 and consists of 16 exons [18]. Four types of repeated sequences are found in the MAPT gene: (a) short interspersed nuclear elements (SINEs), (b) long interspersed nuclear elements (LINEs), (c) DNA transposons, and (d) transposable elements with long terminal repeats (LTRs) [19].

**Figure 2:**
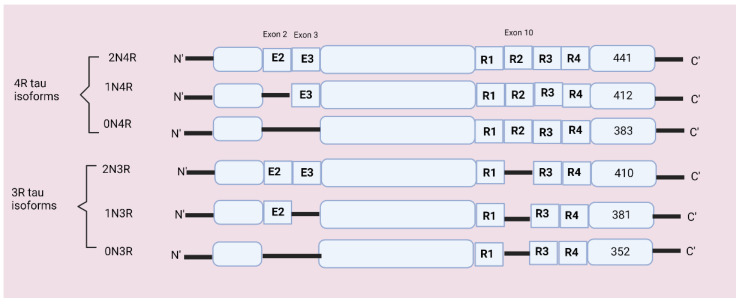
MAPT gene and 6-isoforms structure: The tau gene is located on chromosome 17q21 and contains 16 exons. Six isoforms of tau are generated by alternate splicing of the exons 2, 3, and 10 of the MAPT gene. The N terminal domain, N1 and N2, are produced from exons 2 and 3. These isoforms differ in length from 352–441 amino acids.

In the total of 16 exons, exon 1 acts as a promotor, integral, or constitutive exon includes exons 1, 4, 5, 7, 9, 11, 12, and 13, and alternatively spliced exons include exons 2, 3, and 10 [20]. Alternate splicing of exon 10 forms a three- and four-repeat (3R and 4R) domain at the C terminal and dysregulation in exon 10 can cause neurodegenerative disorders [21]. Alternative splicing of exons 2 and 3 produces 0N, 1N, or 2N inserts at the N terminal [6,22]. The MAPT gene encodes six tau isoforms containing 352–441 amino acids which are expressed in neurons [23]. The six possible tau isoforms are 3R0N, 3R1N, 3R2N, 4R0N, 4R1N, and 4R2N [6,24]. Refer to Figure 2 for isoforms’ structures.

There are six isoforms of tau protein that differ from each other by the presence of either three-repeat or four tubulin-binding domains and by the presence or absence of sequence encoded by exons 2, 3, and 10 [22]. In the normal human brain, the 3R and 4R tau isoforms occur at a 1:1 ratio, while several tauopathies are characterized by the alteration in the ratio of 3R:4R tau [24]. The tau isoform 2 N is weakly expressed in the human brain, and the microtubule-binding region has three (3R: R1, R3, R4) and four-repeat domains (4R: R1, R2, R3, R4) [25]. Each isoform has four parts:

1. N terminal projection domain—Plays an important role in regulating distance among microtubules
2. A proline-rich domain (PRR)—Helps in cell signaling and interaction with protein kinases and contains abundant phosphorylation sites
3. MTBR domain—Contains three or four internal repeats which bind to the proline-rich domain.
4. C terminal domain—Involved in microtubule polymerization because each isoform has specific physiological properties that are expressed differently during brain development [26,27].

The N terminus domain contains two negatively charged inserts, and the proline-rich domain is positively charged and controls phosphorylation. The N terminus and proline-rich domain together are called the projection domain [28]. The interlinkage between the proline-rich region of tau and the microtubule surface helps microtubule stabilization [29]. N terminal has an isoelectric point of 3.8, the proline-rich domain has an isoelectric point of 11.4, and the C terminal has an isoelectric point of 10.8 [22]. Two extended haplotypes, H1 and H2, cover the entire tau gene and are associated with neurodegenerative disease.

*Tau Haplotypes.* The MAPT gene has two haplotypes known as H1 and H2. The H1 haplotypes are predominantly found in all ethnic groups [30]. H1 haplotypes are highly dynamic and composed of single nucleotide polymorphism with several variations [31]. The H1 haplotypes are associated with familial and sporadic neurodegenerative disorders, such as progressive frontotemporal dementia, Parkinson’s disease, corticobasal degeneration, and progressive supranuclear palsy (PSP). Genome-wide association studies (GWAS) also show that the MAPT H1 haplotype is associated with FTD, CBD, and PSP [32]. The H1 haplotype is divided into two subhaplotypes; the H1c subhaplotype is associated with a higher concentration of tau in CSF and several neurodegenerative diseases [33]. In vitro studies suggest that the MAPT H1 haplotype is better in MAPT gene expression than the H2 haplotype [34,35]. MAPT H1 is also associated with the ApoE-4 allele and leads to faster progression from mild cognitive impairment to dementia in patients with both alleles [36].

The H2 haplotypes are primarily found in Europeans and southwest Asians with an inversion of the 900 kb sequence [30,37]. The MAPT H2 haplotypes are linked to a reduced risk of developing a neurodegenerative disorder [38]. The MAPT H2 haplotypes are also linked with hypotonia and a common form of mental retardation called 17q21.31 microdeletion syndrome [39].

## 3. Post-Translational Modification

Post-translational modification occurs in a protein either promptly after translation or after completing its folding and localization [40]. The primary role of PTM incorporation into any protein, including tau, is to alter the structure and regulate the functional diversity [41]. Acosta and colleagues showed that post-translational modifications (PTMs) and their mimetics within tau-paired helical filament(s), nucleating motifs perturb microtubule interactions and the ability of tau to aggregate and thereby contribute to tau’s pathological role in forming oligomers [42]. In a pathological state, tau undergoes multiple post-translational modifications and conformational changes, which are the proteinaceous hallmark of tauopathies [43]. Approximately 35% of amino acids in tau are prone to pre or post-translational modifications; these residues include serine, threonine, tyrosine, lysine, arginine, asparagine, histidine, and cysteine [41]. Human tau protein undergoes various post-translational modifications, including phosphorylation, truncation, nitration, glycation, glycosylation, ubiquitination, and polyamination [44].

*Phosphorylation.* Phosphorylation and dephosphorylation are the major alterations of physiological tau that changes its association with microtubules and likely other mechanisms [29]. Tau is a phosphorylated protein that contains 85 potential phosphorylation sites, which occur at serine (S), threonine (T), and tyrosine [45]. Under a pathological state, phosphorylation of tau is increased, which reduces its affinity for microtubules, resulting in cytoskeleton destabilization, particularly in neurons [46]. Phosphorylation changes proteins’ electrostatics by adding a negatively charged hydrophilic group and resulting in overall hydrophilic protein [41]. The process of phosphorylation of tau is conducted by multiple kinases and phosphatases. Any imbalance in tau kinase and phosphatase activity is believed to cause tau phosphorylation [41]. More than 20 protein kinases can phosphorylate tau protein [47]. These protein kinases are categorized into below groups:

1. The proline-directed protein kinases (PDPK): These protein kinases phosphorylate tau on serine or threonine residues that are followed by a proline residue [47]. The PDPK includes cyclin-dependent kinase 5 (cdk5), mitogen-activated protein kinase, and several stress-activated protein kinases [17]. GSK3-β often comes under the PDPK, but the proline is not always required for phosphorylation by GSK3-β [17].
2. Non-proline directed protein kinases (non PDPK): Tau-tubulin kinases 1 and 2, casein kinases 1 and 2, DYRK1A (dual-specificity tyrosine-phosphorylated and -regulated kinase 1A), phosphorylase kinase, Rho kinase, PKA (protein kinase A), PKB (protein kinase B)/Akt, PKC (protein kinase C) and PKN (protein kinase novel) comes under non PDPKs category [48].
3. Protein kinases specific for tyrosines: Src family kinases such as FYN [49,50].

In their study, Gloria Lee and colleagues reported that tau could be phosphorylated on tyrosine residues (tyr 18) by fyn [51]. Fyn-mediated phosphorylation and interaction between fyn and tau may change its trafficking and cause synaptic impairment due to tau mis-sorting [46].

The equilibrium between the activities of kinase and phosphates determines both the phosphorylation state and biological activity of the tau protein [52]. In vitro study done by Rankin and colleagues showed that the phosphorylation of tau by GSK-3β is sufficient enough to form the tangle-like filament structure [53].

O-GlcNAcylation, a process adding O-GlcNAc (O-linked β-N-acetylglucosamine) to serine and threonine residues [54], plays a protective role against tauopathies [55]. The serine and threonine sites of tau are also affected by O-GlcNAcylation, many known O-GlcNAcylation sites are nearby or found to be overlapping with phosphorylation sites, and thus it has been suggested that these two could regulate phosphorylation positively or negatively. For instance, the inhibition of glycoside hydrolase O-GlcNAcase halts the removal of O-GlcNAc moieties from intracellular proteins that prevent developing tau pathology [56]. Recently, Yin and colleagues’ study showed that SIRT1 member of Sirtuins, a family of signaling proteins, increases the protein level of O-GlcNAcase by C/enhancer binding protein(C/EBPα). SIRT1 negatively regulates the O-GlcNAcylation of tau through mediating O-GlcNAcase (OGA) and O-GlcNAc transferase (OGT) expression in brain-specific SIRT1- knockout mice(SIRT1 floxCre+). They also found that the increased O-GlcNAcylation of tau is accompanied by the enhanced site-specific phosphorylation of tau in the SIRT1 flox/Cre+ brain. Also, the conditional deletion of SIRT1 in mice brains changes the synaptosomal proportion of site-specific phospho-tau and causes dendrites spine impairment and synaptic dysfunction [57].

In the AD brain, dysfunction in glucose metabolism may cause decreased tau O-GlcNAcylation, which in turn, facilitates hyperphosphorylation of tau that leads to neuronal death [58]. Abnormal phosphorylation of tau may cause conformational changes, proteolytic cleavage, and aggregation [59]. Abnormal phosphorylation decreases its affinity for microtubules and causes neuronal and synaptic loss in the brain [49]. Increased kinase activity is not only actively involved in tau hyperphosphorylation but also decreases dephosphorylation [17].

*Truncation.* Post-translational modification truncation is critically involved in the progression of AD due to neurodegeneration and possible misfolding or aggregation [60]. Tau truncation plays a major role in tau aggregation as well as neurodegeneration [61]. In an AD brain, tau is truncated at various sites by a variety of proteases, and proteolytic pieces of tau are prone to aggregate [62,63]. Research indicates that truncation of tau occurs at Asp41 (D421) and Glu391 sites, where tau truncation makes tau more prone to aggregation. However, the truncation of tau in the oligomeric condition is not completely understood in the AD brain [61,63]. Carboxy terminal truncation of tau is carried by caspase-3, whereas amyloid-beta promotes caspase-3 activation in the cases of AD [64]. Recent research suggests that truncated tau of the C terminal spread into the brain and causes neuronal damage and death [63]. Truncated tau also dysregulates synaptic proteome in the presynaptic and postsynaptic compartments [65]. Truncated tau in the presynaptic ending is associated with dysfunction in the stability of the microtubules that could be responsible for the decrease in synaptic vesicles, and postsynaptic ending truncated tau reduced the levels of neurofilaments [65].

*Acetylation.* Acetylation of tau at lysine 280 is a pathological modification that may contribute to tau-mediated neurodegeneration by both elevated losses of normal tau properties (reduced solubility and microtubule assembly) and gain in toxic functions of tau (increased tau aggregation) [66]. Tau acetylation can be initiated by different mechanisms, including histone acetyltransferase p300 (p300 HAT), cAMP-responsive element-binding protein (CREB)-binding protein or auto-acetylation, with sirtuin 1, and histone deacetylase 6 acting to deacetylate tau [67]. Recently Kim and colleagues investigated the effect of acetylation of tau at the K280 site on tau phosphorylation. They transinfected human neuroblastoma cell line SH-SY5Y with p300 acetyltransferase and tau to induce acetylation of tau and found overexpression of p300 acetyltransferase in tau transfected SH-SY5Y human neuroblastoma cells increased acetylation of tau [68]. Acetylation also causes tau pathology by enhancing tau cleavage, preventing ubiquitin binding, and hindering tau turnover [69]. Acetylation of tau is particularly interesting because many lysine residues that are targets for acetylation are also targets for other PTMs, including ubiquitination, SUMOylation, methylation, and glycation [70]. Forty-four lysine residues of tau occur on the longest isoform of tau protein (2N4R), and thus, 10 percent of the protein has the potential to be altered by acetylation [41].

*Ubiquitination:* In the AD brain, PHFs are highly ubiquitinated, and ubiquitination likely plays a vital role in tau filament formation [71]. Ubiquitination of tau occurs on lysine residues and contains seven lysine residues [41]. The process of ubiquitination has three successive stages, each of them involving a different enzyme: (a) an E1 ubiquitin-activating enzyme that catalyzes the ATP-dependent ubiquitin activation, (b) an E2 ubiquitin-conjugating enzyme that activates ubiquitin is transferred through a transesterification reaction, and (c) an E3 ubiquitin ligase essential for catalyzing the formation of the isopeptide bond between ubiquitin’s terminal glycine and the target protein [70]. CHIP, a ubiquitin ligase, enhances the ubiquitination of the tau and also increases tau aggregation [72].

*Methylation:* In both pathological and normal brains, lysine methylation has been detected in extracted tau proteins [70]. The process of methylation is carried out by a class of enzymes called methyltransferases [73]. Tau methylation occurs at s lysine and a few arginine sites [73]. The electrostatics of tau protein changes due to methylation [74]. Funk and colleagues in vitro study on the role of methylation of tau on MTBR suggested that methylation is part of the modification [75].

*SUMOylation:* In tau SUMOylation, a small protein SUMO is post-translationally attached to the target protein. This SUMO protein is enzymatically transferred to the side chain of lysine at the terminal amino group [70]. Tau SUMOylation at K340 reciprocally enhances its phosphorylation and inhibits ubiquitination-mediated tau degradation. They conducted in vitro tubulin polymerization assays in the absence of Tau and the presence of synthetically methylated Tau. It was observed that Tau methylation does not affect the proportion of tubulin polymerization in its methylated state [74].

## 4. Aggregation of Tau Protein

Among above mentioned PTMs, probably not all are directly responsible for tau aggregation [49]. Phosphorylation, glycation, and nitration promote tau aggregation, while the glycation process is involved in tau polymerization [26,76]. In physiological conditions, tau protein is extremely flexible and highly soluble, and most tau filaments are easily able to aggregate [27]. Due to the highly flexible nature of tau protein, and it carries many charges, it tends to interact with many partners in the congested cytosol [77]. It is often believed that aggregation of tau is initiated by hyperphosphorylation of tau [78]. Tau monomers are highly soluble proteins, intrinsically disordered proteins with an open structure ranging from 55 KDa to 74 KDa, and have an increased level of β sheet structure and a reduced level of random coiling [52]. Tau oligomers are primarily composed of monomeric or dimeric subunits of tau protein that can either be hyperphosphorylated or pathologically truncated [79]. Tau oligomers act as intermediate structures that can induce conformational changes in the structure of the tau and attach to the oligomeric structure, resulting in the formation of stacked beta-sheet strands and forming insoluble paired helical filaments. These paired helical filaments then turn into large NFTs [80]. Tau oligomers are the complex structure of two or more molecules of tau protein in a multimeric structure and contain only one protein that is either a hyperphosphorylated or non-phosphorylated protein [81]. In vitro studies suggest that in addition to polyanion, free fatty acid being a molecule with negative charges at several sites increases the possibilities of tau aggregation [82], and tau aggregation occurs once neurofilament and phosphorylated tau are at risk to alter by carbonyl products [83].

*Aggregated Tau and Oxidative Stress*. Oxidative stress is an imbalance between oxidants and antioxidant mechanisms, which causes cell death in neurodegeneration [84]. Oxidative stress, which is mainly caused by an imbalance between reactive oxygen species (ROS), is mainly produced by mitochondria, peroxisomes, and the endoplasmic reticulum [11]. The deposition of ROS in AD is caused by many factors, such as mitochondrial impairment that leads to defects in ETC and the production of ROS [84]. Oxidative stress generates oxidized fatty acids that stimulate in vitro tau polymerization [85]. Long-term mitochondrial stress induces a cytosolic adaptive response, which is responsible for increased tau aggregation [86]. Several kinases, such as glycogen synthase kinase-3 (GSK3), and mitogen-activated protein kinases, including extracellular receptor kinase, p38MAPK, activates by oxidative stress and have the potential to phosphorylate tau and formation of neurofilaments [87,88,89,90]. Extensive studies show that cells with overexpressing tau are more susceptible to oxidative stress [91]. Much evidence suggests that oxidative stress may have a role in abnormal phosphorylation and polymerization, which cause tau aggregates.

## 5. Genetics of Tau (Mutation and Splicing Imbalance)

Tau mutations were first discovered in the late 1990s in inherited frontotemporal lobar degeneration (FTDP-17) families, which include G272V, P301L, V337M, and R406W [92,93]. Researchers identified mutations in the MAPT gene in over 150 families with frontotemporal lobar degeneration [92,94,95]. Familial tauopathy is associated with abnormal phosphorylated tau protein, and studies showed that mutations in the MAPT gene are sufficient enough to induce the formation of pathological tau protein [96]. These mutations could affect the tau at the protein level as well as at the RNA level [97]. At the protein level, K257T, G272V, P301L, V337M, and R406W mutations alter the binding properties of the microtubule, and N279K, N296H, P301S, S305I mutations at the RNA level shifts the splicing towards overproduction of 4R tau [97].

Identification of the mutation in the tau gene in frontotemporal dementia and parkinsonism (FTDP) is linked with chromosome 17, which is directly linked to the impairment of tau and neurodegenerative disease [98,99]. FTDP is inherited as an autosomal dominant condition exhibited by behavioral disturbance, cognitive decline, and motor disturbance [99]. In autopsy analysis of patients suffering from FTDP-17, frontotemporal atrophy with neuron loss, grey and white matter gliosis, and superficial cortical spongiform changes are visible [99]. In tau, exons 9–13 mutations are all pathogenic [99]. Researchers investigated over 50 FTDP-17 families and found more than 11 missense mutations, three base pair deletions, and five splice site mutations in exons 9–13 of the tau gene [98]. All known mutations in the tau gene occurred in the C terminal and affected the exon 9–12, which encodes microtubule-binding repeats [98]. All mutations alter the level of tau isoforms and their ability to bind and promote the microtubule and increase the aggregation of tau into filaments [17]. Several MAPT mutations alter the ratio of 3R and 4R tau isoforms and accelerate tau phosphorylation [46].

## 6. Tau Pathology

Researchers indicate that tau plays a major role in the pathogenesis of tauopathies as a result of studies that observed tau binding with nucleic acid during chromatin remodeling to protect against reactive oxygen species (ROS), stabilize ribosomes, and control the activity of mRNA [100].

In the case of AD, the formation of neurofibrillary tangles emerges from layer II of the entorhinal cortex and disseminates in the limbic and associated areas, finally reaching the hippocampus and neocortex [101]. In tauopathy diseases such as AD, three progressive stages are proposed: (a) preclinical high-risk stage, (b) mild cognitive impairment stage, and (c) disease manifestation stage [29]. The failure of the tau protein to stabilize microtubules leads to the development of AD. The majority of tauopathy patients also show an accumulation of amyloid beta, α-synuclein, or Huntingtin [102].

Abnormally high levels of tau concentration are directly linked with its accumulation in the form of NFTs and PHF in the progression of AD pathogenesis [103]. The process of tau degradation has been done by two different mechanisms, i.e., the ubiquitin-proteasome system and the autophagy-lysosome pathway [103]. The ubiquitin-proteasome system worked as the primary clearance mechanism for pathological tau, while the autophagy-lysosome pathway degraded tau at the late stage of NFTs formation [103]. A major cause of tau deposition is the impairment of its degradative pathway [104].

Hyperphosphorylated Tau also inhibits kinesin-dependent peroxisome transport, making cells vulnerable to oxidative stress, and degenerating affected neurons [91]. Since tau protein is potent in inhibiting the transportation of cellular components in cultured fibroblasts, this condition leads to the reversal of mitochondria to the cell center where the microtubules’ minus ends would be anchored [91]. The absence of mitochondria and ER in the peripheral region of the axons can cause inhibition in glucose and lipid metabolism, ATP production, and loss of Calcium dyshomeostasis [105].

Experimental model studies have provided evidence that mitochondrial impairment could result in pTau, microtubule depolymerization, and NFT-like pathology [106]. Reddy’s lab study also revealed that p-tau interacts with mitochondrial fission protein Drp1 and enhances GTPase Drp1 enzymatic activity, leading to excessive fragmentation of mitochondria and mitochondrial dysfunction in AD [107].

**Figure 3:**
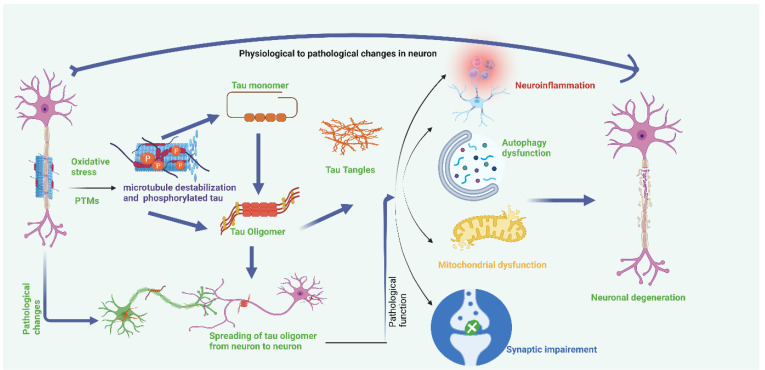
Oxidative stress and post-translational modifications cause tau hyperphosphorylation, which causes instability in microtubules. Microtubule instability causes the formation of tau tangles from the tau monomer and tau oligomer. Accumulation of tau tangles causes neuroinflammation, autophagy dysfunction, mitochondrial dysfunction, and synaptic impairment, which results in neuronal damage. Tau oligomers also spread from neuron to neuron.

Hyperphosphorylation of tau and disassembly of tau from microtubules also induces tau mis-sorting from axons into the somatodendritic compartment, disturbing axonal microtubule integrity and inducing synaptic impairment [108]. Phosphorylation also alters the association of tau with its interacting partners, including cytoplasmic membrane, DNA, and Fyn, disturbing the functions of tau in a range of signaling pathways [109]. Refer to Figure 3.

AD and MCI patients with acetylcholine have demonstrated tau pathology in forebrain neurons [110]. Moreover, before perirhinal cortex pathology, the occurrence of pretangles and tangles in the basal forebrain suggests the formation of tau in the forebrain, and abnormalities in cholinergic neurons occur in early life and increases gradually with the age and development of AD [110]. Another piece of evidence from the study on the tau transgenic mouse model suggests similar observations that tau pathology is found to be participating in cholinergic degeneration [111].

## 7. Role of P-Tau in Alzheimer’s Disease

**Figure 4:**
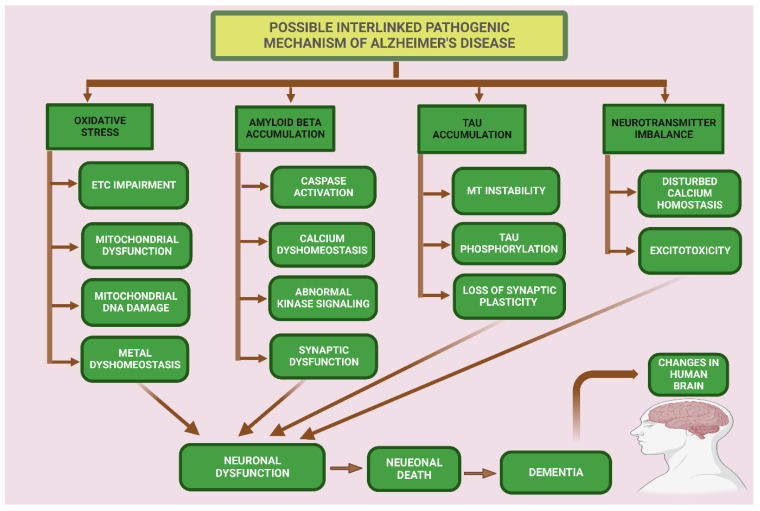
Pathogenic hypothesis for neuronal dysfunction in Alzheimer’s disease. Mitochondrial oxidative stress condition accelerates the electron transport system to generate more reactive oxidative stress and cause oxidative damage in brain cells. Aβ accumulation causes caspase activation of abnormal kinase signaling and synaptic impairment. Caspase activation increases tau phosphorylation. Tau accumulation in cells causes microtubule instability which causes tau phosphorylation. A neurotransmitter imbalance causes disturbed calcium homeostasis and excitotoxicity. Oxidative stress, Aβ accumulation, tau accumulation, and neurotransmitter imbalances result in neuronal dysfunction, which causes neuronal death and leads to dementia.

In AD and other tauopathies, tau is abnormally phosphorylated, and this dysregulation of tau results in neuronal and synaptic loss in the brain. Tau phosphorylation in the AD brain is characterized by at least a three-fold increase in phosphorylation [112]. Three different types of pools of tau are found the in the brains of AD patients: (a) AD tau is most similar to normal tau and is not hyperphosphorylated; (b) AD Phosphorylated tau (AD P-tau) is soluble hyperphosphorylated tau, and (c) paired helical filaments (PHFs)-tau is insoluble and hyperphosphorylated [113]. Tau accumulation results in brain cell damage, but the exact process of tau toxicity is still unclear. In AD, tau also shows a loss of microtubule binding, which is probably due to the hyperphosphorylation at different sites that detach tau from microtubules and cause the breakdown of intracellular traffic, which results in the dying back’ of neurons [114]. Vigo Pelfrey and colleagues study observed that there is an increase of tau in the cerebrospinal fluid, which probably arises from dying neurons [115]. In AD, the mis-sorting of tau from an axonal to a somatodendritic pattern showed that there is a defect in directing tau into the right compartment. This could arise from several mechanisms which could lead to the degeneration of synapses [77]. The protein degradation systems, such as proteasome and autophagy, play important roles in preventing the abnormal deposition of proteins in different cellular compartments, and both degradation systems are compromised in AD [116]. Refer to Figure 4.

## 8. Tauopathies

**Figure 5:**
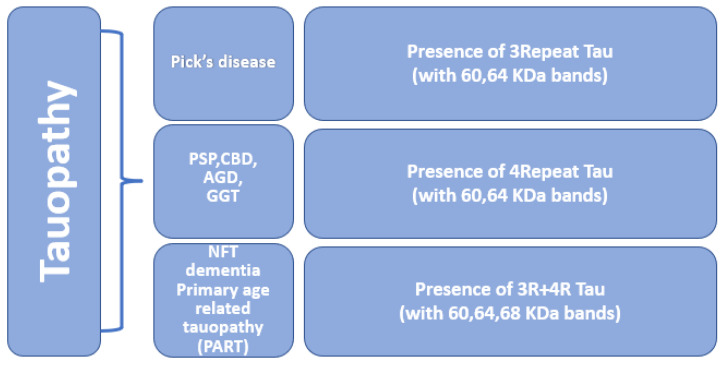
Molecular classification of primary tauopathy. Tauopathies are divided into three categories based on the presence of repeat isoforms. 3-repeat tauopathy is linked with Pick’s disease. The presence of 4-repeat isoforms is linked with PSP, CBD, AGD, and GGT. The presence of both 3-repeat and 4-repeat tau isoforms is associated with primary age-related tauopathy and tangles-only dementia.

Tauopathies are a heterogeneous group of neurodegenerative disorders in which the deposition of abnormal tau proteins is one of the main neuropathological characteristics [117]. Mutation in the MAPT gene is associated with impairment in the normal binding of tau to tubulin and causes accumulation of phosphorylated tau. Abnormal accumulation of tau is a pathological hallmark of many neurodegenerative diseases such as frontotemporal dementia, Pick’s disease, Alzheimer’s disease, argyrophilic grain disease, progressive supranuclear palsy, and corticobasal degeneration [118], refer to Figure 5. In some tauopathies, pathological changes follow a stereotypic pattern and show the spreading of tau pathology [119]. Based on the ratio of 3R and 4R tau isoforms and the presence of two or three major phospho-tau bands in western blotting, tauopathies can be categorized into different categories [9].

*Frontotemporal Dementia*. Frontotemporal dementia is increasingly considered a common form of non-Alzheimer’s dementia, which is clinically characterized predominantly by memory loss, behavioral problems, and frontal and temporal lobar cortical atrophy [120]. There are three clinical features of FTD: (a) behavioral variant frontotemporal dementia, (b) non-fluent variant primary progressive aphasia, and (c) semantic variant primary progressive aphasia, which majorly affects personality and behavior [121]. Histological examination shows loss of large cortical nerve cells and a spongiform degeneration of superficial neutrophils. Post-mortem pathological examinations reveal bilateral atrophy of frontal and anterior temporal lobes and the degeneration of the striatum [122]. Immunohistochemical analysis shows four major types of pathological features: (a) micro-vacuolation without neuronal inclusions, (b) micro-vacuolation with ubiquitinated rounded intraneuronal inclusions in frontal and temporal neocortex and hippocampal dentate gyrus cells, (c) swollen achromatic neuron and transcortical gliosis with rounded intraneuronal inclusions, and (d) micro-vacuolation and tau positive NFTs in neurons and sometimes tangles in glial cells of the cerebral cortical white matter [123]. Three major proteins (i.e., phosphorylated tau, transactive response DNA binding protein 43 [TDP-43], and fused in sarcoma [FUS] proteins) are involved in FTD [124].

Mutations in seven genes, including MAPT, GRN, TARDBP, FUS, VCP, CHMP2B, and C9ORF72, can cause dominantly inherited FTD [125]. Two types of mutation in the MAPT gene are linked with FTD, the first missense mutation alters the amino acid sequence of the encoded tau protein, and the other MAPT mutation alters the pattern of tau alternative RNA splicing [126]. A mutation in exon 12 (E342V) also affects tau at RNA as well as protein levels and in vitro decreases tau’s ability to promote MT assembly and appears to affect the splicing in of exon 10 [127].

*Huntington’s Disease.* Huntington’s disease (HD) is an inherited neurodegenerative disease characterized by progressive dystonia, cognitive decline, chorea, and psychiatric symptoms [128,129]. HD is identified by abnormal deposition of inclusion containing mutant HTT in the brain. HD is primarily linked with protein misfolding and insufficient clearance [129]. HD is an autosomal dominant inherited disease caused by expanded CAG repeat on chromosome 4p16.3. In the Huntington gene, the mutant protein formed by CAG repeat leads to polyglutamine strands at the N terminus. Tau phosphorylation is extensively reported in HD [130,131]. Mutant HTT protein is caused by the expansion that forms oligomers, and globular intermediates, which cause widespread neuronal dysfunction and neurodegeneration in the cerebral cortex and striatum as a result of aggregates [132]. Numerous studies showed the possible role of hyperphosphorylated tau in HD that could contribute to the disease [133].

Indirect interaction between HTT and tau could lead to tau phosphorylation modulation [117]. In HD, tau polymorphism has been associated with the advancement of cognitive decline [133]. Romina Vuono and colleagues examined post-mortem brain samples from patients with HD and investigated the role of tau in the pathology of HD, and found that MAPT haplotypes affect the rate of cognitive decline in a large group of patients with HD, some mutant HTT aggregates co-localized with both the 3R- and 4R-tau deposits, and altered 4R:3R tau isoform ratio in Huntington’s disease brains [132]. David Blum and colleagues’ studies suggested that mutant HTT expression is linked with altered tau function, while in vitro studies suggest that to affect tau hyperphosphorylation, mutant HTT causes changes in its cellular distribution [134].

*Argyrophilic Grain Disease.* Argyrophilic grain disease (AGD) is characterized by the accumulation of argyrophilic and four-repeat (4R-) tau as grains in the medial temporal lobe structures, entorhinal cortex, hippocampus, and amygdala [119,135]. There is no specific diagnostic test for AGD [135]. The main clinical features of AGD can only be diagnosed through post-mortem findings. The pathological features include argyrophilic grains, oligodendrocytic coiled bodies, and neuronal pre-tangles [136]. Ultra-structurally, argyrophilic grains are straight and contain paired helical filaments made up of hyperphosphorylated tau [137].

Many studies confirm that the key protein ingredient of argyrophilic grain and coiled bodies is abnormally hyperphosphorylated tau protein [138]. In both argyrophilic grain and coiled bodies, tau protein shares a high number of phosphorylated sites with NFTs and neuropil threads, including Thr181, Ser202, Thr205, Thr212, Ser214, Thr231, Ser235, Ser396, Ser404, and Ser422 [138]. Whereas AGD lacks tau acetylation [139].

*Amyotrophic Lateral Sclerosis.* Amyotrophic lateral sclerosis (ALS) is known as Lou Gehrig’s disease. In ALS, both upper and lower motor neurons degenerate and die, which causes an inability to control muscle movement and eventually leads to total paralysis [140]. Studies indicate that TAR DNA binding protein-43, protein aggregates, and hexanucleotide repeat expansion in C9orf72 are the main causes detected in ALS patients [141]. In ALS, ubiquitinated protein deposits in degenerating motor neurons. The SOD1, TDP-43, and FUS genes are commonly muted genes. The molecular mechanism by which motor neurons degenerate and die is still ambiguous [142]. Many molecular and cellular activities have been implicated in patients with ALS, including mitochondrial dysfunction, axonal transport, toxic protein aggregation, protein degradation, oxidative stress, hypermetabolism, prion-like spreading, ribonucleic acid (RNA) metabolism defects, and RNA toxicity [143].

Alternation in the tau protein metabolism is widespread and the most consistent pathology of ALS [144]. In vitro studies suggest that the change in tau metabolism is caused by pathological phosphorylation at site Thr 175, which is linked with the activation of GSK3β, phosphorylation of Thr 231 tau, and the development of cytoplasmic inclusions with a shoot-up rate of cell death [144].

*Pick’s Disease*. Pick’s disease is characterized by widespread degeneration of white matter, degeneration of numerous Pick’s bodies, formation of Hirano bodies, granulovacuolar degeneration of neurons, and occurrence of neuronal chromatolysis [145,146]. Pick’s bodies are made up of vesiculated endoplasmic reticulum, neurofilament, and paired helical filament that is mostly phosphorylated tau [147,148].

Immunohistochemical studies indicate that tau phosphorylation in Pick’s bodies is linked with stress kinases SAPK/JNK and p38 [149,150]. Deposition of phosphorylated tau in Pick’s disease is linked with oxidative stress, with the possibility of oxidative stress causing activation of p38 and deposition of phosphorylated tau [151]. The p38 pathway is an intracellular signaling pathway that is triggered by cellular stress (i.e., oxidative stress, hypoxia, and apoptotic signal) and finally leads to a change of transcription [152,153].

*Progressive Supranuclear Palsy.* Progressive supranuclear palsy is a clinical syndrome characterized by supranuclear palsy (PSP), postural inability, and mild dementia. Neuropathologically, PSP is referred to as the deposition of neurofibrillary tangles [154]. In PSP, tau protein, after losing its ability to bind MT, become resistant to proteolysis [155]. Different post-translational changes may be implicated in the aggregation of tau, but glycation and trans-glutamination are major mechanisms that are involved in PSP [155]. In PSP, severe neurotransmitter changes, damage to the dopaminergic nigrostriatal cycle, and cholinergic damage in most of the area are very consistent [156].

Researchers find that the over-excitable nature of extracellular regulated kinase 1 and extracellular regulated kinase 2 (ERK1 and ERK2) is associated with tau hyperphosphorylation in PSP [157]. Stressed-activated protein kinases (p38SAPK) are also triggered off in PSP along with upstream regulator MAK-7 (mitogen-activated kinase 7) [151]. An imbalance between the tau isoform and post-translation modification of the tau protein results in the deposition of abnormal tau in PSP [158].

Polymorphisms in the tau gene may increase the risk of developing PSP because a polymorphic dinucleotide repeat in the intron between E9 and E 10 of the tau gene has been associated with PSP. MAPT mutation in exon 10 and intron 10 is also seen in PSP [159].

*Globular Glial Tauopathy.* Globular glial tauopathy (GGT) is an occasional type of four-repeat tauopathy characterized by a neuronal accumulation of hyperphosphorylated, truncated, oligomeric, and abnormally conformed 4FR tau in neurons and glial cells (oligodendroglia and astrocyte) with globular inclusions [160]. GGT is classified into three subtypes [160].

Type 1: Prefrontal and temporal cortices distribution of pathology.

Type 2: Both the motor cortex and the degeneration of the corticospinal tracts.

Type 3: Involves a combination of prefrontal, temporal motor cortex, and corticospinal tracts.

Primary Age-Related Tauopathy (PART). PART pathology often notices in the brain of elderly people [161]. According to recent research, the PART entity could cause cognitive decline in the absence of AD [162]. It is a neuropathological confirmation indicated by tau NFTs in the absence of amyloid plaque pathology [163]. In PART, increased levels of NFTs are linked with expeditious cognitive decline [163].

Cortico-Basal Degeneration (CBD). CBD is a gradual neurodegenerative disease that often co-exists with asymmetrical parkinsonism with a variable combination of ideomotor apraxia, myoclonus, rigidity, and dystonia linked with the presence of an alien limb phenomenon [164]. Neuropathological signs and symptoms of CBD include tau inclusions in neurons and glia, extensive thread-like pathology, and astrocytic plaques in both white and grey matter [165]. Tau protein is considered to be a main key factor in CBD pathology and is characterized by the deposition of insoluble tau enriched in the (4R tau) four-repeat [165,166]. Mutation in the MAPT gene could display clinical and pathological features associated with CBD [167,168].

Chronic Traumatic Encephalopathy (CTE). CTE is defined as a progressive neurodegenerative disease caused by the consequence of a closed head injury that might be a single injury or repetitive one with no treatment and diagnosis [169]. Repeated traumatic brain injuries cause damage to the axon and altered membrane permeability. The ionic imbalance leads to calcium influx, the successive release of caspases and calpains provokes tau phosphorylation, conformational change in the structure of tau, and aggregation [170]. CTE is a tauopathy and is characterized by the accumulation of hyperphosphorylated tau in the form of NFTs, astrocytic tangles, and neurites cluster around the cortex in the blood vessels, predominantly in the sulcal depth [171]. The progressive post-traumatic degeneration may involve extracellular and intercellular spreading of tau throughout the brain [171].

## 9. Tau-Aβ Interaction

An increase in the interaction between P-tau and Aβ was found to be associated with a damaged neuronal condition that could cause cognitive deficits in AD patients [172]. Amyloid beta aggregates enhance the hyperphosphorylation of tau by mediating the activation of CDK-5 [173]. Jin and colleagues isolated Aβ oligomer directly from the cortex of a LOAD patient and observed that Aβ oligomer is sufficient to accelerate hyperphosphorylation of tau at AD-relevant epitopes and cause instability in microtubule cytoskeleton followed by abnormal neuronal processes [174]. Caspases are cysteine aspartate proteases that directly cleaved APP during apoptosis and lead to the formation of elevated beta-amyloid peptides [175].

In vitro study done by Chris and colleagues found that tau is proteolyzed by multiple caspases at a highly conserved aspartate residue (Asp421) in its C terminus, and its proteolysis strike to introduce nuclear events of the apoptosis. They also report that caspase cleaves tau has been shown to generate truncated protein and accelerate tau aggregation and suggested that Aβ peptides accelerate pathological tau in neurons by stimulating caspase cleavage of tau and formation of proteolytic products with the tendency of polymerization kinetics [175,176]. Amadoro and colleagues’ study results from both cellular and transgenic animal models indicate that tau protein is essential for Aβ-induced neurotoxicity [177].

Experimental studies on tau derived from postmortem cortical extracts and brain interstitial fluid of tau-transgenic mice, as well as human AD cortices, have demonstrated that the presence of Aβ accelerates the production of a rare soluble species of high-molecular-weight (HMW) hyperphosphorylated tau, which appears to be a competent substrate for the intercellular spread [178]. Although Aβ and tau protein becomes toxic through different mechanisms, In vitro, animal, and human studies have found a direct link between Aβ and tau in causing toxicity in AD. Lars and colleagues suggested three possible modes of interaction between Amyloid beta and tau that are a) Aβ drives tau pathology, b) synergistic toxic effects of Aβ and tau, and c) tau may mediate Aβ toxicity [179]. The hypothesis states that neurons become more vulnerable to Aβ induced damage in the postsynaptic dendrites in the presence of higher tau concentrations within the dendrites [179].

## 10. Tau and Synapse

Synaptic gain is fundamental to healthy brain functions [180]. The word “synapse,” which means “coming together,” was introduced in 1897 by Foster and Charles Sherrington and is defined as the mode of nexus between two neurons. The concept of the synapse emerged from consideration of how muscles are contracted and how locomotion is affected by muscle contraction. Synapse is the site of connection where two nerve cells transfer electric nerve impulses (action potential). Synapses are required for the transmission of the nerve impulse from one neuron to target another neuron [1]. In the nervous system, a synapse is a structure that allows a neuron to pass an electrical or chemical signal to another cell. Synapse connections are the main location from where abnormal or diseased tau spreads to healthy neurons [181].

Synapses are extremely plastic and can change their strength because of their activity or by the activity in another pathway [182]. According to many researchers, synaptic plasticity plays a central role in understanding the mechanism of learning and memory. Synaptic plasticity, both in excitatory and inhibitory synapses, is an important function of the brain that depends on the postsynaptic calcium release [183]. Synaptic loss and dysfunction are primary characteristics of many neurodegenerative diseases, including AD [180]. Synapse dysfunction is the first event in AD progression and is fundamental to the pathophysiological progression of AD, resulting decrease in cognitive function [184]. Synaptic dysfunction is considered an endpoint incident of neurodegenerative diseases [185]. Although the exact mechanism of synaptic loss and dysfunction is not clearly understood, research indicates that reduced synaptic activity and density are one of the major events that happen before neuronal loss [186].

Tau is predominantly present in the axon compartment of neurons. Under pathological conditions, tau detached from an axonal compartment of the microtubule abnormally localizes into a presynaptic and postsynaptic compartment of the neuron and induces synaptic dysfunction in various ways, such as reduced mobility, the release of a presynaptic vesicle [187,188]. Tau is also found in Golgi bodies, the rough endoplasmic reticulum, autophagic vacuoles, neuronal plasma membranes, and within synapses responsible for protein trafficking and secretion [189,190]. Tau can arbitrate toxicity by interacting with synaptic vesicle protein and preventing vesicle release [80].

Tau primarily interconnects within synapses with actin, microfilament, postsynaptic density protein 95, NMDAR, and Kinases FYN [191]. In AD, hyperphosphorylated tau also deposits in the somatodendritic compartment of neurons, and abnormal localization of hyperphosphorylated tau in dendritic spines may cause disturbance in the trafficking of glutamate receptors and synaptic dysfunction [192]. Tau damage to the synaptic compartment is the major cause of synaptic loss and can spread from one neuron to another neuron throughout the brain [193].

## 11. P-Tau and Mitochondria

Mitochondria are double membrane-bound organelles within the cell and are known as the powerhouse of the cell. Mitochondria produce cellular energy for the cell in the form of adenosine 5′-triphosphate through oxidative phosphorylation [14]. Mitochondria are essential to fulfilling high-energy demands, regulation of calcium buffering, neurotransmission, generation of membrane potential, and sufficient delivery of mitochondria in neurons [194]. In addition to ATP formation, mitochondria also perform other activities, such as the formation of reactive oxygen species, calcium regulation, and apoptosis. The brain is very susceptible to oxidative damage because it consumes a high rate of oxygen and has an increased level of polyunsaturated fatty acid which gradually increases the level of redox transitional metal ion and low antioxidant level [195,196]. Damaged mitochondria are eradicated by an autophagic activity called mitophagy [197]. Research suggests that the brain is a high-energy-consuming body part, so even a small change in the energy metabolism may cause a disturbance in the central nervous system, so energy impairment is one of the initial and consistent features of AD [61,198].

A mitochondrial cascade hypothesis for AD was introduced in 2004 by Russell Swerdlow [199]. The cascade hypothesis states that mitochondrial dysfunction and other provoked events are a consequence of mitochondrial dysfunction that causes late-onset AD [200]. Aggregated tau and overexpression of hyperphosphorylated tau damage the axonal transport, which causes the abnormal distribution of mitochondria [29]. Disturbance in the equilibrium mitochondrial dynamics of fusion and fission results in an imbalance of mitochondria in neurons [201]. Another abnormal function of mitochondria is the synthesis of neurosteroids. These neurosteroids play a major role in the regulation of neuronal function as they act as an allosteric modulator of neurotransmitters of receptors (e.g., gamma-aminobutyric acid, n-methyl-D-aspartate). The initial process of steroidogenesis begins in the mitochondria with cholesterol transfer from the cytosol to the mitochondrial matrix and its alternation into pregnenolone that is then converted into other neurosteroids either in mitochondria or endoplasmic reticulum [202]. AD patients exhibit oxidative stress in mitochondrial microglia, producing reactive oxygen species and loss of mitochondrial membrane potential by a different process [201].

## 12. P Tau-Induced Inflammation

Inflammation occurs in most neurodegenerative diseases. Neuroinflammation is a complex inflammatory response within the brain or spinal cord against an inflammatory challenge that causes the activation of microglia and astrocyte, release of cytokines, chemokines, reactive oxygen species, and secondary messengers [203]. Reactive microglia, reactive astrocytes, and inflammatory molecules are also observed around neurofibrillary tangles (NFTs) in AD brains [204]. Tauopathies also show reactive gliosis and enhanced inflammatory molecules such as complementary protein and cytokines, which are collectively known as neuroinflammation [205]. All the cytotoxic entities work in synergism to induce neurotoxicity that leads to neurodegeneration [206].

Microglia can play a variety of functions; microglial function is dependent upon their activation status and the encountered pathologic event, which may be either neurotoxic or neuroprotective [207]. Under the physiological state, microglia are active to play a role in the maintenance of homeostasis, neuroprotection, and neuro repair by releasing growth factors such as brain-derived neurotrophic factor (BDNF) and transforming growth factor (TGF) β with ramified morphology. While in a pathological state, microglia become activated, proliferate, and change from a ramified to an amoeboid, macrophage-like morphology [198]. Astrocytes are predominantly found in the brain and perform many functions in the CNS, such as modulating synaptic formation, maintaining neuronal homeostasis via metabolic support, controlling the blood–brain barrier, and acting as chemosensors, thus maintaining homeostasis (regulation of energy balance, blood pH and Na+ concentration) [208]. The deposition of abnormally phosphorylated tau in astrocytes is known as aging-related tau astrogliopathy (ARTAG) [209].

Activated microglia, astrocytes, and increased concentration of proinflammatory molecules are also pathological characteristics of tau pathology found in brain regions affected by tau pathology [210]. Microglia can cause beneficial as well as harmful effects, depending on the microenvironment and cytokines involved [211]. In tauopathies, tau aggregates accumulate in both neurons and astrocytes [212]. Impairment of BBB found in tauopathies is driven by chronic neuroinflammation [213]. The inflammatory processes enhance structural alteration in capillaries, such as fragmentation, thickening, atrophy of pericytes, accumulation of laminin in the basement membrane, and increased permeability of blood vessels to plasma proteins [213]. Tau-induced activation of glial cells enhances the expression of endothelial adhesion molecules and increases the transport of leukocytes across BBB [214]. Tau accumulation has been associated with increased production of IL-1β, IL-6, TNF-α, and MCP-1, all of which are implicated in BBB dysfunction [215].

There are two types of stereotypical hypotheses for the anatomical progression of different tauopathies: (a) affected brain regions and cell subpopulations are selectively vulnerable to tau pathology, secondly (b) the spreading of tau pathology from one interconnected brain region to another region [216]. Sarah C. Hopp and colleagues suggested that microglia play a complex role in the propagation of tau pathology. According to their study, microglia take up tau, are not capable of fully degrading it, and in turn, release tau that could accelerate aggregation in recipient cells [217]. Histopathological and positron emission tomography (PET) neuroimaging studies showed that topical changes in the phenotype of microglia correlate with cognitive decline and or tau pathology in AD and other tauopathies [218].

## 13. Therapeutics of P-Tau

The development of therapeutics for AD was primarily centered on Aβ, but tau has now gained more attention in the last few years due to the clinical trial failure of Aβ targeting therapies. Several studies suggest there are many new possible approaches to block tau toxicity using: like direct reduction of pathological tau, tau post-translational modification inhibition, inhibition of tau propagation, and microtubule stability of tau [102]. Hyperphosphorylated tau is considered a potential therapeutic target for AD because of its close association with neurodegeneration and cognitive impairment [64].

A wide range of therapeutic approaches is currently under investigation. Possible tau-aimed therapies are immunotherapies in preclinical trials. Most of the therapeutic approaches include molecules that enhance microtubule stability against loss of tau function in pathology. Other therapeutic approaches include (a) rectification of autophagic flux, (b) harmonizing ubiquitin-proteasome system component, and (c) response of endoplasmic reticulum stress system [64,219,220]. Several tau therapeutic approaches are proposed, including active and passive immunotherapies, inhibition of protein kinase, inhibition of tau aggregation, and tau silencing by antisense oligonucleotides [220]. Several different animal models currently suggest that p-tau epitopes enhance antibody response and are competent to promote tau clearance [221]. This section discusses passive and active tau therapeutic vaccinations, tau modification, antisense therapeutics, natural therapeutics, and medicines approved by the Food and Drug Administration.

Passive Vaccines for Tau Immunotherapy. Passive immunotherapy includes the administration of monoclonal antibodies. Several researchers suggest that anti-tau antibodies block tau aggregation seeding and reduce tau pathology in transgenic mice. Semorinemab—an IgG4 monoclonal antibody—targets the N terminal of tau and has high affinity and specificity, and binds to all isoforms of tau [222]. BIIB076—an IgG1 antibody categorized as a pan tau antibody—treats a monomeric and fibral form of tau protein [223]. UCB0107—another monoclonal antibody—targets the central region of tau protein to inhibit tau seeding capacity to prevent the spread of neurofibrillary tau protein JNJ63733657, an IgG1 antibody recognizes the microtubule-binding site of tau, and it has a high affinity at residue 217 for phosphorylated tau [224].

Active Immunotherapy (Tau Vaccines). Active tau immunization is an appealing strategic tool because it produces constant antibodies in small amounts [102]. The active AADvac-1 vaccine induces specific antibodies aimed at three or four conformational epitopes of tangles to form tau protein with an exposed microtubule-binding repeat domain to prevent spreading, prevent tau aggregation, and promote tau clearance [225]. The ACI-35 liposome-based vaccine incorporates a synthetic peptide antigen anchored into a lipid bilayer to activate the immune system to produce antibodies designed to selectively target the pathogenic form of tau.

Targeting Tau Modification. Targeting post-translational modification changes, alone or in combination, has the potential to prevent tau aggregation and improve the normal function of tau protein [64]. Lysine acetylation also promotes tau aggregation in addition to phosphorylation. The use of lysine acetylation inhibitors might be a possible therapeutic approach for AD [221].

Antisense Oligonucleotide Therapy (ASO). Antisense oligonucleotide therapy is based on synthetic oligonucleotides designed to bind to RNA by Watson–Crick base pairing [226]. Antisense oligonucleotide nucleotides are 15–30 nucleotides in length and can be single-stranded or double-stranded [227]. After the fulfillment of toxicology studies, Bennett, Krainer, and colleagues initiated a phase I clinical trial using treated mice that targeted the tau gene in mild AD patients [226]. This clinical trial found that direct targeting of tau gene expression with an antisense oligonucleotide therapy reduces tau seeding activity in brain lysates [226].

Natural Products as Tau Targeting Compounds. Currently, there is a limited number of drugs available to treat AD, and most of these only treat symptoms. Due to the rapid increase in the number of AD cases worldwide, there is a need for alternative therapies [2]. Ayurvedic herbs are a rich source of antioxidants, anti-inflammatory agents, and neuroprotective, anti-amyloidogenic, and adaptogenic agents [228,229]. Some ayurvedic herbal plants are useful in treating neurological disorders.

Ashwagandha (Withania somnifera), cinnamon (Cinnamomum verum), turmeric (Curcuma longa), brahmi (Bacopa monnieri), and many other herbal plants show anti-tau effects in recent studies. Turmeric contains curcumin, which is a diarylheptanoid that stimulates the production of anti-inflammatory IL-4 cytokines and reduces Aβ and tau concentration in mice with Aβ overexpression [230]. Curcumin also reduces the pro-inflammatory cycle by blocking the proliferation of TNF-α, IL-1β, and other pro-inflammatory cytokines in astrocytes and microglia [231].

In addition to plant-based ayurvedic herbal extracts, the following bacterial- and fungal-derived compounds are reported to have anti-tau efficacy: anthraquinones, daunorubicin, and Adriamycin derived from the bacterium *Streptomyces peucetius* [232]. In vitro and cells, the compounds daunorubicin and Adriamycin inhibit tau aggregates and dissolve paired helical filaments [232]. Table 2 shows a summary of current trials underway on tau-targeted therapeutics.

## 14. Conclusions and Future Directions

The microtubule-associated tau protein in the brain helps to stabilize axonal microtubules. However, the abnormal accumulation and aggregation of phosphorylated tau lead to the disease state in AD and other tauopathies, but precise underlying mechanisms of normal tau and/or phosphorylated tau regulation in healthy and diseased conditions are still unclear. Phosphorylation and dephosphorylation of tau protein cause alterations in the physiological roles of this protein. In a pathological state, tau undergoes multiple post-translational modifications and conformational changes, which are the hallmarks of AD and other tauopathies like Pick’s disease, Alzheimer’s disease, argyrophilic grain disease, progressive supranuclear palsy, corticobasal degeneration. Phosphatase and kinase activity plays a major role in abnormal phosphorylation, which leads to the aggregation of tau. In AD, abnormally hyperphosphorylated tau aggregates occur in neurons, which leads to synaptic loss and neuronal damage in the brain. However, the role of hyperphosphorylated tau in the formation of NFTs is a subject of debate.

Pathological tau is an attractive target for the treatment of AD and other tauopathies. Most of the recent drug discoveries to reduce tau pathology are based on inhibiting tau phosphorylation and preventing or slowing tau production. Approaches to reduce the abnormal hyperphosphorylation of tau include the inhibition of one or more tau kinase activities, activation of one or more tau phosphatases, and the enhancement of *O*-GlcNAcylation of tau. Other tau-based drug discovery includes immunotherapies like tau vaccines and therapeutic monoclonal antibodies. But there are many limitations owing to which tau-based drugs still need more research to be safe and effective. Moreover, a better understanding of tau protein misfolding, accumulation, and immune responses triggered by its propagation during aging and neurodegeneration would be significant for designing and developing efficacious therapies for Alzheimer’s disease and other tauopathies.

## Figures and Tables

**Table 2:** List of tau-targeted current trial therapeutics.

Name	Synonym	Therapy Type	Target	Condition	Status
LMTM	TRx0237, LMT-X, Methylene Blue, Tau aggregation inhibitor (TAI)	Small Molecule	Tau	Alzheimer’s Disease, Frontotemporal Dementia	AD (Phase 3) FTD (Phase 3)
TPI 287		Small Molecule	Tau	Alzheimer’s Disease, Corticobasal Degeneration, Progressive Supranuclear Palsy	AD (Inactive) CBD(Inactive) PSP (Phase 1)
BIIB080	IONIS-MAPTRx, ISIS 814907	DNA/RNA-based	Tau	Alzheimer’s Disease	AD (Phase 1)
Semorinemab	RO7105705, MTAU9937A, RG6100	Immunotherapy (passive)	Tau	Alzheimer’s Disease	AD (Phase 2)
BIIB076	NI-105, 6C5 huIgG1/l	Immunotherapy (passive)	Tau	Alzheimer’s Disease	AD (Phase 1)
Bepranemab	UCB0107, UCB 0107, Antibody D	Immunotherapy (passive)	Tau	Progressive Supranuclear Palsy, Alzheimer’s Disease	PSP (Phase 1) AD (Phase 2)
JNJ-63733657	-	Immunotherapy (passive)	Tau	Mild AD	AD (Phase 2)
AADvac1	Axon peptide 108 conjugated to KLH	Immunotherapy (active)	Tau	Alzheimer’s Disease, Progressive Nonfluent Aphasia	AD (Phase 2) PNA (Phase 1)
ACI-35	VAC20121	Immunotherapy (active)	Tau	Alzheimer’s Disease	AD (Phase 2)

## Data Availability

Not applicable.

## Abbreviations

4R-	Four-repeat
AChE	Acetylcholinesterase inhibitors
AD	Alzheimer’s disease
AGD	Argryophilic grain disease
ALS	Amyotrophic sclerosis
APP	Amyloid precursor protein
ASO	Antisense oligonucleotide therapy
ASO	Antisense oligonucleotide therapy
ATP	Adenosine 5′-triphosphate
Aβ	Amyloid beta
BBB	Blood–brain barrier
CBD	Corticobasal degeneration
CNS	Central nervous system
DMT	Disease-modifying therapy
DNA	Deoxyribonucleic acid
EOAD	Early-onset Alzheimer’s disease
FDA	Food and Drug Administration
FTD	Frontotemporal dementia
FTDP	Frontotemporal dementia and parkinsonism
GABBA	Gamma-aminobutyric acid
GRN	Gene and progranulin
GWAS	Genome-wide association studies
IFN	Interferon
LOAD	Late-onset Alzheimer’s disease
LTP	Long-term potentiation
MAPT	microtubule-associated protein tau
MCI	mild cognitive impairment
mRNA	messenger ribonucleic acid
NFT	Neurofibrillary tangles
NFT	neurofibrillary tangles
NMDA	N-methyl-D-aspartate
OGA	O-GlcNAcase
OGT	O-GlcNAc transferase
PD	Parkinson’s disease
PDPK	proline-directed protein kinases
PHF	Paired helical filament
PTM	Post translational modification
RNA	ribonucleic acid
ROS	reactive oxidative species
TGF	transforming growth factor
TNF	tumor necrosis factor
U.S.	United States
